# Rubinstein-Taybi Syndrome: A Case Report

**DOI:** 10.1155/2012/483867

**Published:** 2012-09-06

**Authors:** A. P. Münevveroglu, B. B. Akgöl

**Affiliations:** Department of Pedodontics, Faculty of Dentistry, Istanbul Medipol University, Fatih, 34093 Istanbul, Turkey

## Abstract

Rubinstein-Taybi syndrome or Broad Thumb-Hallux syndrome is a genetic disorder characterized by facial dysmorphism, growth retardation, and mental deficiency. A seven-year-old girl had come to the Department of Pedodontics, Istanbul Medipol University, Faculty of Dentistry, Turkey, with a complaint of caries and bleeding of gingivae. The patient was mentally retarded. Extraoral features revealed distinctive facial appearance with a broad fore head, hypertelorism, broad nasal bridge, and beaked nose. Intraoral features observed were talons cusps in the upper lateral incisors, carious teeth, and plaque accumulation. Since the patient was mentally retarded, the dental treatment was done under GA. The treatment plan and dental management of this patient are discussed in this case report.

## 1. Introduction

Rubinstein-Taybi Syndrome (RTS:OMIM 180849), or Broad Thumb-Hallux syndrome, was initially described by Michail et al. in 1957 [1]. In 1963, Rubenstein and Taybi reported on seven cases of this syndrome [2], which included a group of congenital anomalies consisting of short, broad thumbs and great toes, psychomotor retardation, highly arched palates, and histories of recurrent respiratory infections and particular facial abnormalities [3–5]. It is caused by either a microdeletion at 16p13.3 or mutations in the CREB-binding protein (CREBBP or CBP) or EP300 gene (at 22q13) [4–7].

The incidence of the syndrome has been estimated to be 1 in every 300,000 newborns [7]. There is an equal male and female incidence [8–10]. 

The main distinctive features most commonly linked to this syndrome are downward sloping palpebral fissures, broad thumbs and halluces, growth retardation and psychomotor developmental delay, typical facial dysmorphism, hypertelorism, a prominent forehead, and mental disability [9–11]. 

One intriguing phenomenon is the fact that RTS patients are prone to develop tumours. These tumours show a pattern of neural and developmental origin, and neuroblastoma, medullablastoma, oligodendroglioma, meningioma, seminoma, odontoma, choristoma, and polimatrixtomas were the reported tumours in RTS patients [3, 12, 13]. 

Congenital anomalies of cardiovascular system are also described such as ventricular septal defect, patent ductus arteriosus, coarctation and stenosis of the aorta, and pulmonic stenosis [3, 14, 15].

Oral manifestations of this syndrome include limited mouth opening, a pouting lower lip, retro/micrognathia, a high arched and narrow palate, cleft uvula and palate, and rarely a cleft upper lip. Dental abnormalities occur in 67% of individuals with RTS and can include hypodontia, maintenance of deciduous teeth, talon cusps, and enamel hypoplasia. An increased rate of caries and periodontal disease has been reported in these patients. However periodontal disease has not always been associated with this syndrome [7, 11, 16]. Characteristics of RTS are described in Table 1.

Dental treatment is generally complicated due to difficulties in managing the patient's behavior. In most patients with this syndrome, it is necessary to carry out the dental treatment under sedation or general anesthesia. It is important to know that these patients might have upper respiratory obstruction during sleeping or sedation because of the anatomical characteristics of their maxillofacial region [16–18]. 

## 2. Case Report

A 7-year-old girl accompanied by parents reported to the faculty of Dentistry, Department of Pedodontics, Medipol University, Turkey, with a complaint of caries and bleeding of gingiva. She is the second child of a remote consanguineous couple. Her old sister had no signs of dental anomalies. The patient was born at term, weighing 2 kg 850 g. 

**Figure 1:**
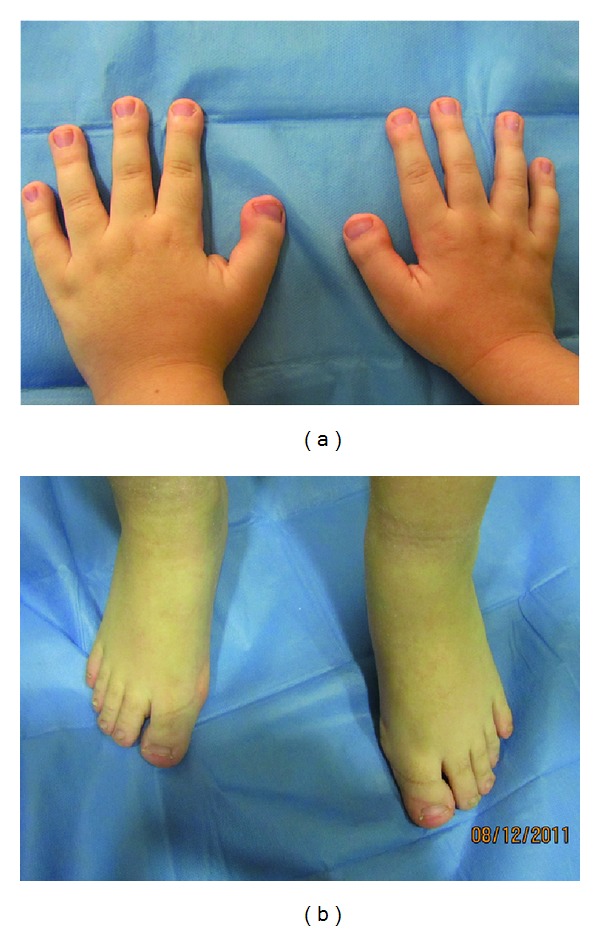
Hands and feet in RTS. Broad thumbs, broad terminal phalanges were seen.

**Figure 2:**
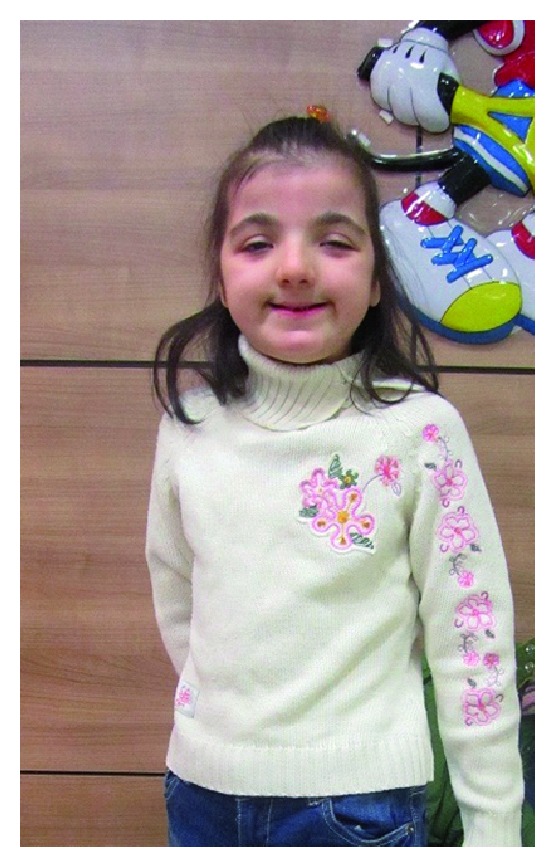
Facial characteristics include slanting palpebral fissures and beaked nose with nasal septum.

The following clinical extraoral findings were observed: short stature with broad thumbs, mental retardation, down slant of the palpebral fissures, strabismus and simple ear (Figures 1(a), 1(b)). The nose has a beaked appearance, broad fleshy bridge, deviated septum, and short low columella (Figure 2) and she had cardiac abnormalities.

**Figure 3:**
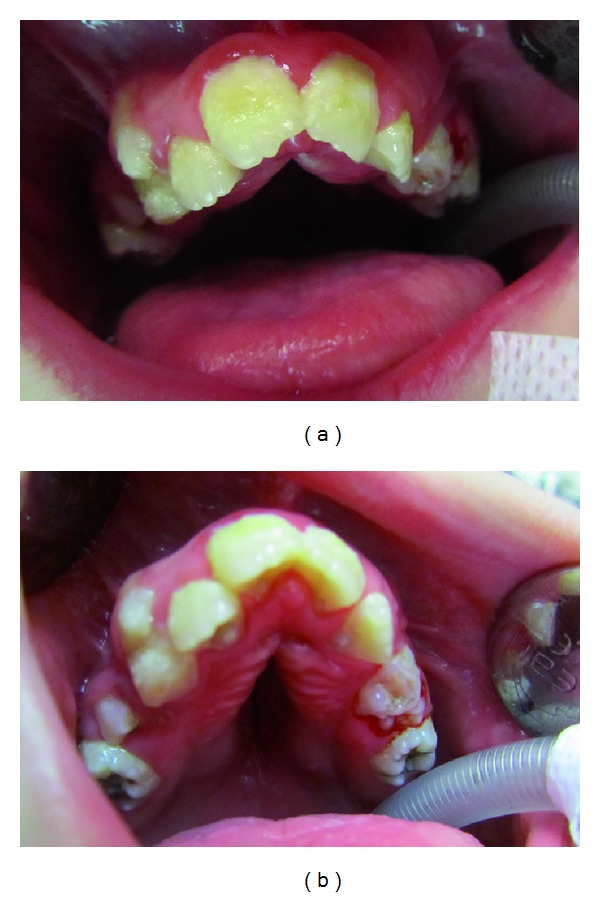
(a) Intraoral findings were a small mouth and malocclusion. (b) In intra-oral findings, high-arched and cleft palate was seen. And upper molar teeth had deep dentinal carious lesions.

**Figure 4:**
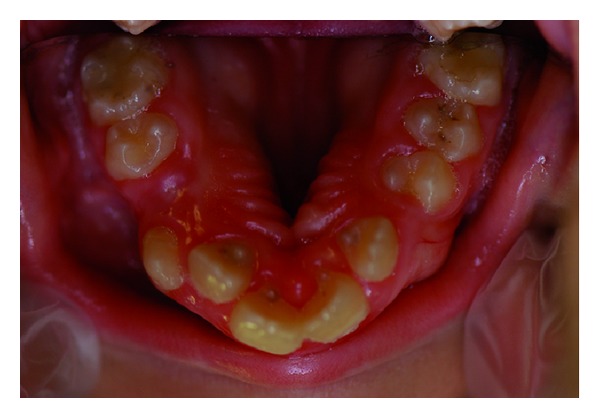
Talon cusps were present on maxillary central and lateral incisors.

**Figure 5:**
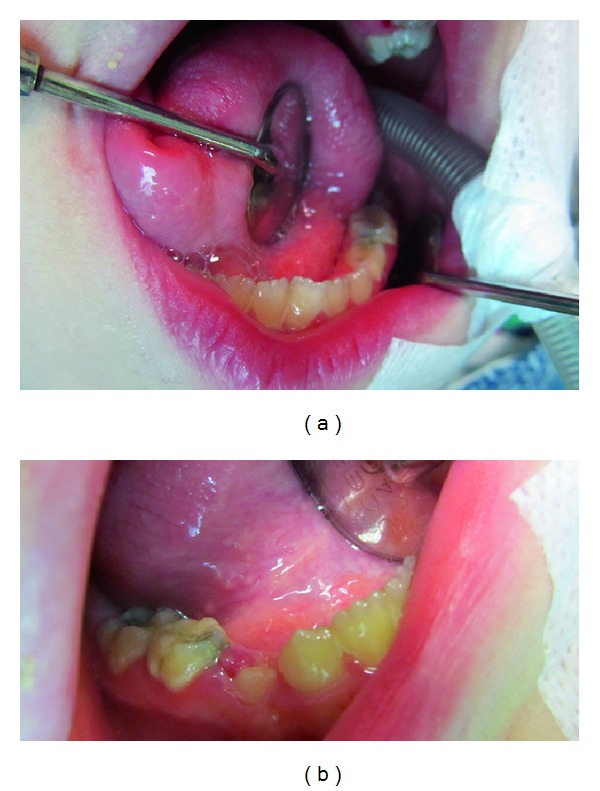
Lower molar teeth had deep dentinal carious lesions.

Intra-oral findings were high-arched and cleft palate, a small mouth and malocclusion (Figures 3(a) and 3(b)). Talon cusps were present on maxillary central and lateral incisors (Figure 4). The patient also had carious lesions (Figures 3(a), 3(b), 4, 5(a), 5(b)). Upper and lower molar teeth had deep dentinal carious lesions (no. 16, no. 26, no. 36, no. 46, no. 75, no. 85). Heavy calculus and plaque deposition could be noted on all teeth. The marginal gingiva was severely swollen and probing resulted in profuse gingival bleeding.

After the complete clinical examination and investigation, the following dental treatment plan was derived.

1. Oral prophylaxis and topical fluoride application.
2. Cavity preparation and composite resin restoration (no. 16, no. 26, no. 36, no. 46, no. 75, no. 85).
3. Extraction of no. 53, no. 64.
4. Recall follow-up sessions for every 3 months.

Due to difficulties in the patient's behavior and her inability to cooperate during dental treatment because of her mental disability, the treatment was performed under general anesthesia. 

Detailed oral hygiene instructions were given to her mother who provided routine care for the patient and the patient recalled follow-up sessions every 3 months. However, because of her incoordination about brushing teeth; evidence of unimproved dental hygiene was observed and instead of this no new caries were found. Because of plaque deposition on all teeth, periodontal treatment consisted of scaling, and oral prophylaxis and topical fluoride application were performed under sedation after 6 months later. 

## 3. Discussion

The 7-year-old girl described in this paper was diagnosed with RTS associated with a microdeletion at 16p 13.3 chromosome. She had cardiac abnormalities, which are frequently found in 24–38% cases [7, 16, 19].

RTS is a rare multiple congenital anomaly syndrome [20]. Intraoral findings of the patient with RTS were reduced oral opening, a narrow palate, malocclusion, gingivitis, and caries. It was reported that patients with RTS have an increased rate of caries (15% to 36%) because of their poor oral hygiene, which was similar to our patient [7, 12, 21]. Malposition and crowded teeth are present in 62% of patients [7, 22]. In this patient, upper and lower molar teeth had deep dentinal carious lesions (no. 16, no. 26, no. 36, no. 46, no. 75, no. 85). Heavy calculus and plaque deposition could be noted on all teeth. The marginal gingiva was severely swollen and probing resulted in profuse gingival bleeding, and malposition was one of, important problems in our patient.

In most patients with this syndrome, it is necessary to carry out the dental treatment under sedation or general anesthesia. It is important to know that these patients might have upper respiratory obstruction during sleeping or sedation because of the anatomical characteristics of their maxillofacial region [16–18]. The treatment of the patient was performed under general anesthesia by anesthesiologist. 

Freitas et al. [19] reported the periodontal and immunological status of a 14-year-old female patient with RTS and possible association between syndrome and periodontal disease was reported in the paper. In this paper, heavy calculus and plaque deposition could be noted on all teeth. The marginal gingiva was severely swollen and probing resulted in profuse gingival bleeding.

Prospects for future good oral and dental status in this patient are questionable because of her extreme lack of cooperation. For this reason, she needs a longer followup.

## Figures and Tables

**Table 1:** Characteristics of RTS.

Orthopedic	Broad thumbs, first toes, short stature, vertebral abnormalities
Eye	Strabismus, refractory errors, ptosis, coloboma, ptosis, cataracts, nystagmus
Cardiac	Congenital heart defects
Dental	Crowding teeth, malocclusion, multiple caries, hypodontia, hyperdontia, talon cusps
Tumors	Meningioma, neuroblastoma, medullablastoma, oligodendroglioma, seminoma
Skin	Keloids
Sleep apnea	Obstructive sleep apnea
